# Reply to ‘Comment on “Who needs family meals? The association between shared meals and dietary quality among Finnish children, fathers and mothers”’

**DOI:** 10.1017/S0007114526106576

**Published:** 2026-06-28

**Authors:** Henna Vepsäläinen, Reetta Lehto, Eva Roos, Carola Ray, Maijaliisa Erkkola

**Affiliations:** 1 Department of Food and Nutrition, University of Helsinki, PO box 66, 00014 Helsinki, Finland; 2 Folkhälsan Research Center, Topeliuksenkatu 20, 00250 Helsinki, Finland

Dear Editor,

We highly value the opportunity to respond to the Letter to the Editor by Dr Shatavari Kulshrestha^(1)^. This letter concerns our recently published article, ‘Who needs family meals? The association between shared meals and dietary quality among Finnish children, fathers and mothers’^(2)^ and discusses some of its strengths, limitations and implications. We sincerely appreciate the insightful comments as well as the recognition of the article’s contribution to our understanding of family mealtime patterns. The points raised in the letter are very sharp-witted, and we are glad to expand on these ideas below.

## Fathers’ role in the family food environment

We agree with the importance of considering fathers and their meal-sharing practices within the family food environment. As argued in a recently published special issue, there is a need for a shift towards a perspective focused on the parenting behaviours fathers engage in with their children^(3)^. We hope that by studying family meals and dietary quality among father–child dyads, our article has provided some useful evidence on such parenting behaviours. As we strive towards the UN’s sustainable development goal #5 ‘Gender equity’, a considerable emphasis is legitimately put on empowering women and girls. However, in certain contexts, such as the family food environment, attention should be focused on men. Family meals provide fathers with a perfect opportunity to engage in both cognitive and socioemotional caregiving practices. Through food-related questions and explanations, they stimulate children’s understanding and learning, while routines, affectionate interaction, waiting, and turn-taking at the dinner table support children’s emotion regulation and social competence.

## Challenges in capturing preschool-aged children’s diets

We fully recognise and acknowledge the limitations that challenge the interpretation of our findings. In our discussion section, we addressed the selection bias and the exclusion of preschool meals when calculating the Healthy Food Intake Index (HFII) for the children. We noted that even though this led to a probable underestimation of HFII among the children, which hinders comparison with mothers and fathers, it is unlikely that this systematic error would have affected the explored associations. In Finland, preschool meals are guided by recommendations^(4)^ and included in the daycare fee, and thus, parents cannot directly influence the food provided at preschools. To more comprehensively capture diets of preschool-aged children within this context, we call for the development of FFQs that draw on data obtained from multiple informants (fathers, mothers and preschool personnel) to produce a reliable overview of the whole diet. Researchers should also pay more attention to family diversity. For example, some children may live with their parents on alternate weeks, making it extremely important to acquire food consumption data from both parents.

### Conclusions and future directions

We are grateful for the thoughtful discussion points, which have highlighted several areas for future focus, and hope that other research groups interested in gender equity within the family context also consider these in their future work. We could not agree more on the importance of examining parental modelling, family communication and screen/media use simultaneously to better understand mealtime dynamics and the mechanisms through which shared meals contribute to healthier diets. However, to be able to study families, not just mothers and children, we recommend inviting both parents to participate in studies. Researchers must also begin to consciously plan more attractive recruitment methods that encourage fathers’ participation^(5)^. Considering fathers as full-fledged parents, not as auxiliary supports to mothers, helps us get closer to gender equity by distributing caregiving responsibilities more equally between genders. At the same time, it fosters father–child relationships by creating opportunities for meaningful interaction and shared daily life.
